# Chemical-Mediated Targeted Protein Degradation in Neurodegenerative Diseases

**DOI:** 10.3390/life11070607

**Published:** 2021-06-24

**Authors:** Soonsil Hyun, Dongyun Shin

**Affiliations:** Gachon Institute of Pharmaceutical Science, College of Pharmacy, Gachon University, Yeonsu-gu, Incheon 21936, Korea; hobois@gachon.ac.kr

**Keywords:** neurodegenerative disease, protein degradation, drug design, ubiquitin-proteasome system, autophagy, protac

## Abstract

Neurodegenerative diseases, including Alzheimer’s disease, Huntington’s disease, and Parkinson’s disease, are a class of diseases that lead to dysfunction of cognition and mobility. Aggregates of misfolded proteins such as β-amyloid, tau, α-synuclein, and polyglutamates are known to be among the main causes of neurodegenerative diseases; however, they are considered to be some of the most challenging drug targets because they cannot be modulated by conventional small-molecule agents. Recently, the degradation of target proteins by small molecules has emerged as a new therapeutic modality and has garnered the interest of the researchers in the pharmaceutical industry. Bifunctional molecules that recruit target proteins to a cellular protein degradation machinery, such as the ubiquitin–proteasome system and autophagy–lysosome pathway, have been designed. The representative targeted protein degradation technologies include molecular glues, proteolysis-targeting chimeras, hydrophobic tagging, autophagy-targeting chimeras, and autophagosome-tethering compounds. Although these modalities have been shown to degrade many disease-related proteins, such technologies are expected to be potentially important for neurogenerative diseases caused by protein aggregation. Herein, we review the recent progress in chemical-mediated targeted protein degradation toward the discovery of drugs for neurogenerative diseases.

## 1. Introduction

Many neurodegenerative diseases are associated with protein aggregates. Misfolded proteins aggregate into a β-sheet structure, which is a major phenomenon of protein-misfolding diseases (PMDs) [1]. As a defense mechanism against misfolded protein aggregates, cells maintain homeostasis through two main modes of action: (i) refolding of misfolded proteins by molecular chaperones, and (ii) elimination of aggregated forms of pathogenic proteins as the first approach to alleviating neurodegenerative diseases. Decreases in these defense systems promote the deposition of aggregates leading to neurodegenerative diseases. The major neuronal proteins that cause PMDs include tau, α-synuclein, huntingtin, and β-amyloid.

## 2. Protein Aggregates and Neurodegenerative Diseases

### 2.1. Tau

The tau proteins are abundant in neurons and play a role in maintaining the stability of microtubules in axons as the major microtubule-associated proteins (MAPs) [2]. The accumulation of aggregated tau is associated with synaptic dysfunctions, in which tau localization is abnormally shifted from axons to the somatodendritic compartment. Intracellular aggregates of tau, called neurofibrillary tangles, are found in patients with Alzheimer’s disease (AD). Tau proteins become hyperphosphorylated and aggregate into neurofibrillary tangles [3]. Tauropathies, neurodegenerative disorders characterized by the formation of neurofibrils of hyperphosphorylated tau, can also occur in atypical parkinsonian syndromes [4].

### 2.2. α-Synuclein

Parkinson’s disease (PD) is a progressive nervous system disorder that affects the motor system [5]. It is caused by the degeneration of dopaminergic neuronal cells damaged by Lewy bodies, which are aggregates found in the cytoplasm of neurons. The key protein involved in Lewy bodies is an aggregate of α-synuclein. Lewy bodies affect various intracellular targets, including synaptic function [6], as α-synuclein regulates the mobility of synaptic vesicles and, consequently, neurotransmitter release.

### 2.3. Huntingtin

Huntington’s disease (HD) is characterized by uncontrolled movements and cognitive deficits [7]. HD is an inherited progressive disorder caused by a CAG repeat coding for polyglutamine in the huntingtin protein, eventually forming a β-sheet amyloid structure [8]. Inclusion bodies formed by huntingtin are present in regions of the brain that degenerate [9]. Although the presence of huntingtin aggregates is not restricted to regions of the brain, the toxicity of the aggregates is limited to neurons in certain brain areas, such as the cortex and caudate [10].

### 2.4. β-Amyloid

AD is the most prevalent neurodegenerative disease and is characterized by amyloid beta (Aβ) deposition [11]. It is a progressive illness associated with loss of memory, task performance, speech, and recognition of people and objects. The disease is caused by two kinds of protein aggregates: (i) extracellular aggregates known as neuritic plaques composed of the Aβ peptide, which is derived from proteolytic processing of the amyloid precursor protein, and (ii) intracellular aggregates of the MAP tau [12]. These aggregates are known to be toxic to cells, although the mechanism of aggregation is only partially understood. Before forming insoluble fibrils, the pathogenic proteins aggregate into soluble toxic oligomers. The oligomers expose hydrophobic surfaces, thus disturbing the phospholipid bilayer [1].

## 3. Protein Degradation Machinery in Cells

**Figure 1:**
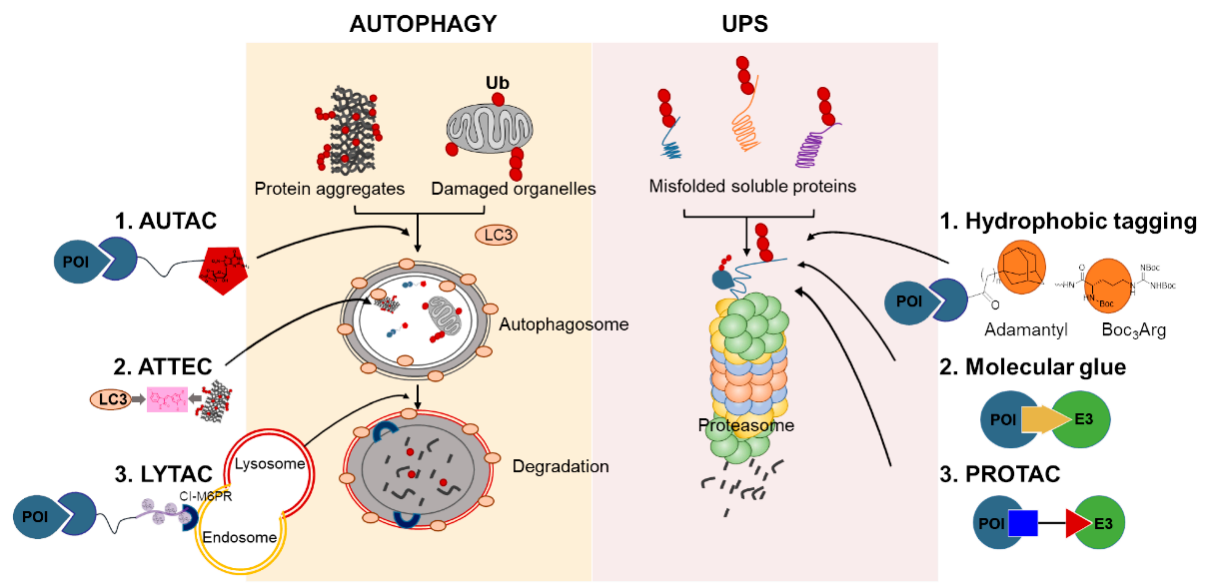
Cellular protein degradation pathways and chemical-mediated targeted protein degradation methods. Ub, ubiquitin; UPS, ubiquitin–proteasome system; LC3, Microtubule-associated proteins 1A/1B light chain 3B; AUTAC, autophagy-targeting chimeras; ATTEC, autophagosome-tethering compound; LYTAC, lysosome-targeting chimaeras; Cl-M6PR, cation-independent mannose 6-phosphate receptor; POI, protein of interest; E3, E3 ubiquitin ligase; PROTAC, proteolysis-targeting chimera.

The main cellular quality control systems for proteins and organelles, the ubiquitin–proteasome system (UPS) and autophagy, form an interconnected quality control network based on biophysical parameters and compartmentalization [13]. These systems are initially known to function in recycling cytoplasm to generate macromolecular building blocks. To achieve homeostasis, cells evolved to have dynamic and self-regulating quality control processes to adapt to new environmental conditions and prevent prolonged damage. Ubiquitination triggers protein degradation through the 26S proteasome system. Ubiquitin, a 76-amino-acid polypeptide, can be covalently attached to a target protein to produce monoubiquitinated or polyubiquitinated products, controlled by multilayered, reversible enzymatic cascade reactions [14]. The eight amino groups (M1, K6, K11, K27, K29, K33, K48, and K63) of ubiquitin provide possibilities for generating the “ubiquitin code” [15], with diverse functional consequences. Meanwhile, autophagy primarily deals with larger cytosolic structures such as protein complexes, cellular aggregates, organelles, or pathogens within the lysosome/vacuole, and the resulting macromolecular constituents are recycled. The best-characterized form of autophagy is macroautophagy, in which the substrates are sequestered within the cytosolic membrane compartment termed the phagophore (an autophagosome) (Figure 1) [16].

In the neuronal cells, the regulation of protein is particularly important since the protein turnover is important for the synaptic plasticity and memory [17]. The UPS regulates the majority of the proteins involved in the postsynaptic response. Regarding the neurodegenerative protein aggregates, it is observed that protein aggregates result from decreased degradation process not increased synthesis [18]. Under the neurodegenerative proteinopathies, neuronal cells are prone to fail to clear aberrant proteins. Although the basic UPS features are similar between neurons and other eukaryotic cells, little is known about how UPS function in specific neurons and subcellular compartments. Therefore, as our understanding of neuronal degradation continues to advance, novel therapeutic approaches should be developed to remove pathogenic aggregates by enhancing proteosomal degradation in neuronal cells.

## 4. Chemical-Mediated Targeted Protein Degradation Methods

Increasing interest is being shown in bifunctional small molecules directing druggable protein targets to specific ubiquitin ligases for polyubiquitination, to mark a pathogenic target protein for degradation, with the subsequent degradation providing therapeutic benefits. Since Crews’ group pioneered the development of bifunctional hybrid molecules composed of a target protein ligand and an ubiquitin E3 ligase ligand in 2001, the proteolysis-targeting chimera (PROTAC) approach has become a promising technology for target protein regulation through a degradation strategy. Protein degradation has been primarily focused on oncotargets thus far. Expanding the indication toward other therapeutic areas is still awaited. For example, proteins involved in neurodegenerative diseases are challenging targets because only one aggregation modulator, methylene blue, is currently being evaluated in clinical trials [19]. As the accumulation of misfolded proteins is a main cause of neurodegenerative diseases, several strategies have been developed for removing misfolded proteins using small-molecule tagging based on the mechanisms of eukaryotic protein quality control systems (Figure 1).

### 4.1. Hydrophobic Tagging

A Food and Drug Administration-approved anticancer drug, fulvestrant (Faslodex, AstraZeneca), is identified to degrade the transcription factor estrogen receptor α (ERα) [20]. The mechanism of this drug is degradation of target proteins by selective estrogen receptor downregulators (SERDs). The hydrophobic tagging (HyT) technology was inspired by the observation that SERDs induce structural changes that result in increased hydrophobic patches. The surfaces of the hydrophobic patches are recognized by the protein quality control machinery, UPS, which induces the degradation of proteins. The attachment of hydrophobic tags, such as adamantyl or Boc_3_Arg, to ligands triggers the unfolding of the target proteins and consequently initiates the protein homeostasis regulatory systems. The general approaches for HyT have been developed on the basis of the mode of action of target protein degradation.

### 4.2. Molecular Glues

Another method of drug-induced protein degradation is the use of molecular glue degraders. These molecules facilitate the interaction between the target protein and a ubiquitin ligase complex, triggering the degradation of the target protein. In the early 1990s, P. Schrieber first described a molecular glue that can stabilize the interaction of two different proteins (e.g., cyclosporin A and FK506). These macrocyclic molecules became known as molecular glues, showing immunosuppressive functions, and synthetic molecular glues/degraders including lenalidomide have been shown to induce target protein adhesion with ubiquitin E3 ligases, resulting in the degradation of target proteins [21]. The major difference of molecular glues from PROTACs is that molecular glues are small molecules that enhance two protein interactions, enabling ubiquitination of the proteins of interest (POIs).

### 4.3. PROTACs

PROTACs are heterobifunctional molecules that degrade a POI. The formation of a ternary complex between the POI and E3 is promoted by the PROTAC composed of the POI ligand and E3 ligase-recruiting ligand. As the first generation of PROTACs, peptidic PROTACs provided the first proof of concept for this technology. Since the small-molecule-based PROTAC targeting the androgen receptor was developed by Crew’s group, a dramatic increase in the use of the PROTAC technology has been reported. Drug resistance in anticancer therapy and immunotherapy is an emerging problem. The PROTAC technology is a promising approach for developing more potent drugs to overcome the problem of drug resistance, owing to the novel mechanism of action that lowers the target protein levels by using lower concentrations of drugs, which are recycled, than those of conventional drugs. The first oral PROTACs to be evaluated in clinical trials are ARV-110 for prostate cancer and ARV-471 for breast cancer from Arvinas [22].

### 4.4. Autophagy-Targeting Chimeras, Autophagosome-Tethering Compound, and Lysosome-Targeting Chimeras

Autophagy is another major intracellular degradation process for maintaining cellular homeostasis. Novel targeted-protein degradation approaches have emerged related to autophagy, the lysosomal degradation pathway. Along with PROTACs, Takahashi et al. developed autophagy-targeting chimeras (AUTACs) [23], which are heterobifunctional molecules conjugated with autophagy-inducing small molecules, as an emerging modality in drug discovery. They identified *S*-guanylation that induces selective autophagy, adapted from the same process as used by cytoplasmic group A *Streptococcus* through the endogenous 8-nitroguanosine 3′,5′-cyclic monophosphate, which exhibits a correlation with K63 polyubiquitination. They used *S*-guanylation as a tag that induces protein substrates for selective autophagy by using chimeric molecules, AUTACs, consisting of a guanine unit and a specific ligand to a POI.

Other approaches using autophagy have been reported such as autophagosome-tethering compound (ATTEC) and lysosome-targeting chimaeras (LYTAC) [24]. ATTEC molecules bind to both the POI and the autophagosome protein LC3, which combines the POI to the autophagosome and consequently induces autophagy. On the other hand, LYTAC is a hetero-bifunctional molecule that targets extracellular and membrane-associated proteins using conjugates that utilize the endosome/lysosomal pathways by binding both a cell-surface lysosome-shuttling receptor and the POIs [24].

## 5. Targeted Protein Degradation for Neurodegenerative Diseases

### 5.1. Tau

**Figure 2:**
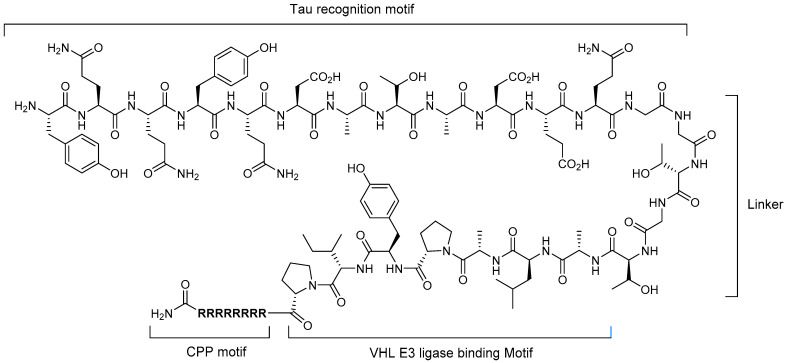
Structure of a peptide-based PROTAC targeting tau protein.

Much effort has been directed toward the elimination or inhibition of tau protein synthesis because tau protein is one of the major pathological proteins in AD. In 2016, Chu et al. reported the first targeted protein degradation system for tau protein using a peptide-based PROTAC compound [25], which consists of four motifs: (i) tau recognition motif, (ii) linker, (iii) E3 ligase-binding motif, and (iv) cell-penetrating peptide (CPP) motif. To select the optimal tau recognition peptide, three known peptides were evaluated and the peptide corresponding to the YQQYQDATADEQG sequence was determined to be the best in their study. Two E3 ligases, Skp1-cullin-F box (SCF) ligase and the von Hippel–Lindau (VHL) tumor suppressor protein, with binding peptide sequences of DRHDS(p)GLDS(p)M and ALAPYIP, respectively, were analyzed. The results showed that VHL is superior to SCF. With a short linker sequence (GSGS) between the tau-binding motif, E3 ligase-binding motif, and polyarginine CPP, the most active PROTAC compound, TH006, was established, which has a 32-amino-acid sequence (Figure 2). TH006 was demonstrated to successfully penetrate into cells and induce tau protein degradation by enhancing polyubiquitination by VHL E3 ligase. Moreover, in an AD transgenic mouse model, it was proven to reduce the neurotoxicity of Aβ through TU005-mediated lowering of the tau protein level.

**Figure 3:**
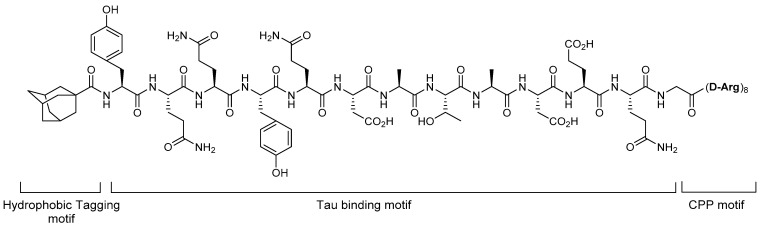
Structure of HyT-Tau-CPP.

The HyT technology was demonstrated to be effective in tau protein degradation. In 2017, Gao et al. reported the degradation of tau protein by an HyT degrader adopting a hydrophobic tag [26,27]. They designed the tau protein degrader by tethering a tau-binding motif to a hydrophobic adamantyl tag at the N-terminal and poly-D-arginine CPP motif. In this HyT-Tau-CPP compound (Figure 3), the linker was not considered and YQQYQDATADEQG was chosen as the tau protein-binding sequence. To investigate the cellular degradation of tau protein by HyT-Tau-CPP, the authors incubated tau-EGFP-overexpressing Neuro2a cells with the compound at increasing concentrations and different times. The western blot analysis of tau protein and flow cytometry assay showed that HyT-Tau-CPP decreased the tau protein in dose- and time-dependent manners. Further, to prove whether the degradation of tau protein by HyT-Tau-CPP is mediated by the UPS, a co-treatment experiment with a proteasome inhibitor, MG132, was performed. The results showed no degradation of the target protein, which indicated that HyT-Tau-CPP degraded the tau protein through the UPS.

**Figure 4:**
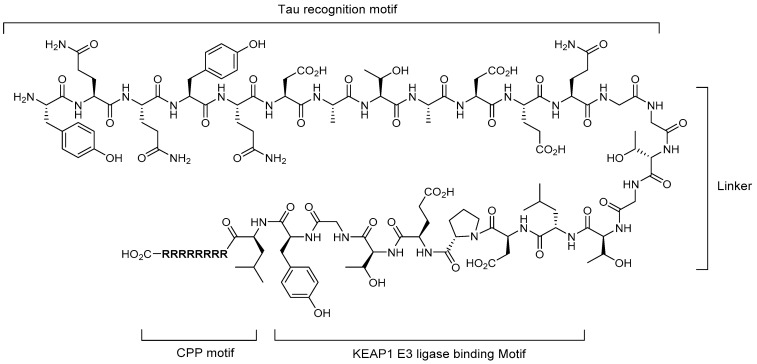
Structure of Tau-Keap1-CPP PROTAC as a tau degrader.

In 2018, Lu et al. reported a peptide-based PROTAC adopting a Keap1 E3 ligase-binding motif, which targets tau protein as the POI [28]. Keap1 (Kelch-like ECH-associated protein 1) is one of the adapter proteins in the Cul3-RING ubiquitin ligase complex and is well known because the Keap1-Nrf2 pathway has cell-protecting functions against various xenobiotic and oxidative stresses that result in neurodegenerative diseases and cancers [29]. In this study, the authors used the Keap1 E3 ligase as a PROTAC technology platform and attempted to prove its potential applications. The overall construct of Tau-Keap1-CPP PROTAC is similar to that previously reported in 2016 [16], in which the VHL E3 ligase-binding motif was adopted. The only difference was that the Keap1-binding motif (LDPETGEYL) was adopted instead of the VHL E3 ligase-binding motif (Figure 4).

For the Tau-Keap1-CPP PROTAC, the *K_d_* values of Keap1 and tau protein were measured using isothermal titration calorimetry assay and determined to be 22.8 and 763 nM, respectively. The designed PROTAC molecules were demonstrated to degrade the cellular tau protein in dose- and time-dependent manners. Through a co-treatment experiment of the proteasome inhibitor MG132 and Keap1 siRNA silencing, the degradation was also confirmed to proceed through the UPS. The value of this study was that Keap1 E3 ligase was demonstrated to be used for the PROTAC technology, broadening the PROTAC platform, while identifying that the binders of Keap1 were peptides.

**Figure 5:**
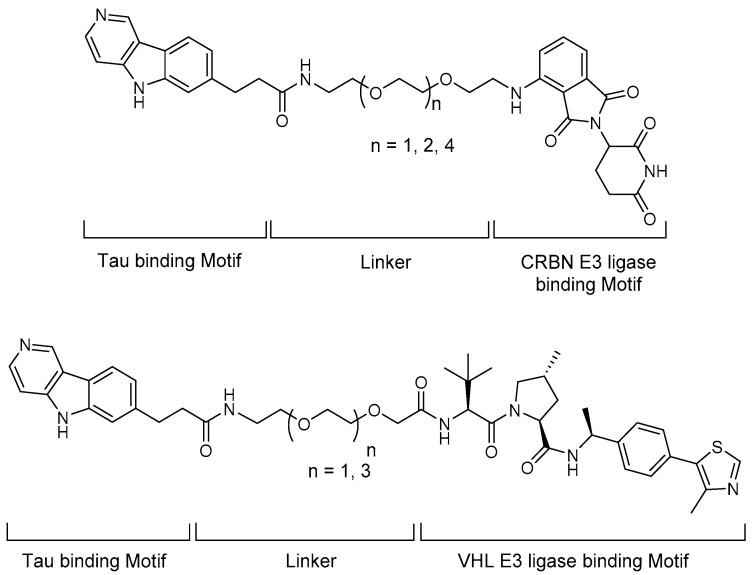
Structures of small-molecule Tau degraders.

In 2019, the first all-small-molecule PROTAC as a tau protein degrader was introduced and briefly reviewed in a “Patent Highlight” article by R.B. Kargbo (Figure 5) [30]. The original report was titled “Compounds for Tau protein degradation” [31]. In this article, six key Tau-targeting PROTACs were selected and the structures were shown. A pyridoindole moiety was introduced as a Tau-binding motif, small-molecule cereblon (CRBN) and VHL binders were implemented, and the two moieties were tethered through polyethylene glycol (PEG)-based linkers. In the degradation experiment using human Tau-P301L and Tau-A152T neurons, hyperphosphorylated and total tau proteins were successfully eliminated by the developed PROTACs. To verify the potential clinical applications of the compounds, an in vivo pharmacokinetic study was performed in mice, which demonstrated several favorable pharmacokinetic parameters, with B/P values of 1.2 and 1.1 based on C_max_ and AUC_0-last_.

**Figure 6:**
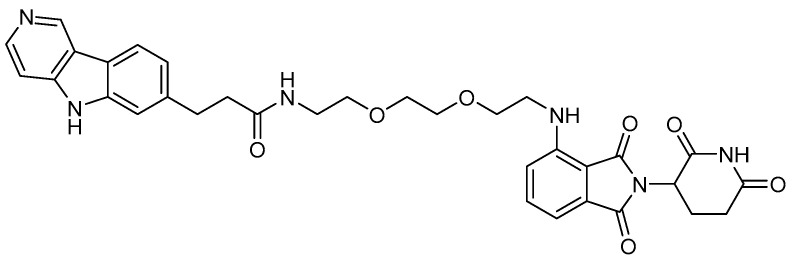
Structure of the tau degrader QC-01-175.

More detailed tau degradation data and biological results of heterobifunctional molecules were reported by M.C. Silva et al. in 2019 [32]. In their article, the degrader compound named QC-01-175 was established to bind CRBN E3 ligase and tau protein to induce ubiquitination of tau protein and proteasomal degradation (Figure 6). The degradation activity was investigated using frontotemporal dementia neuronal cell models, and the results showed the specific degradation of aberrant tau protein. The degradation pathway of this PROTAC compound was confirmed to be the UPS through a comparative degradation experiment via co-treatment of the degrader with/without the autophagy inhibitor bafilomycin A1 and the proteasome inhibitor carfilzomib.

### 5.2. Huntingtin

**Figure 7:**
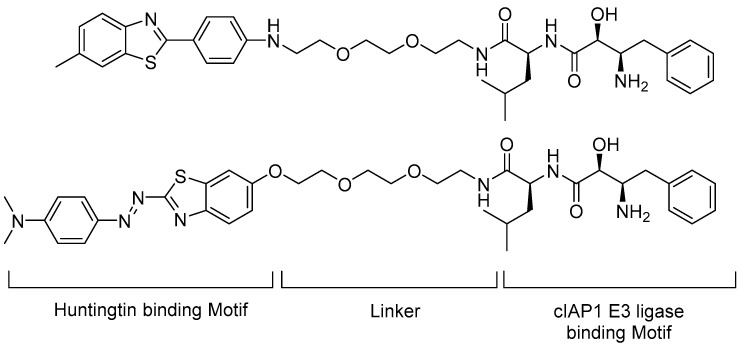
Structures of cIAP1 ligand, bestatin methyl amide BE04 conjugated to BTA (top, Htt-PROTAC 1), or PDB (bottom, Htt-PROTAC 2).

Small-molecule PROTACs have been shown to induce degradation of huntingtin in fibroblasts from HD patients. Tomoshige et al. designed huntingtin-PROTACs (Htt-PROTACs) in which 6-methyl-2-phenylbenzo[d]thiazole and (*E*)-2-(4-(*N*,*N*-dimethylamino)phenyldiazenyl)benzo[d]thiazole were introduced as the targeting ligand (warhead moiety); the dipeptidyl bestatin analogue of cellular inhibitor of apoptosis protein 1 (cIAP1) was adopted as an E3 ligase binder and the two moieties were tethered by PEG-based linkers (Figure 7) [33,34].

A degradation experiment was performed using fibroblast cells from two patients with HD. Htt-PROTAC 1 and 2 significantly reduced the mutant Htt (mHtt) aggregate level at 10 μM concentration without cytotoxicity. An in vitro direct binding experiment was performed with aggregates of 62Q peptides, which showed that the compound interacted with 62Q aggregates [34].

As an extension of the scope of degradation, the authors demonstrated that the discovered compounds also removed other neuronal polyglutamine-containing disease-related proteins, including the polyglutamine-expanded proteins ataxin-3 and ataxin 7, which cause autosomal-dominant neurodegenerative disorders such as spinocerebellar ataxia types 3 and 7. The mutant proteins contained cross-β-sheet structures. By targeting these structures, Htt-PROTAC 1 and 2 were demonstrated to induce the degradation of the proteins in concentration- and time-dependent manners [34].

**Figure 8:**
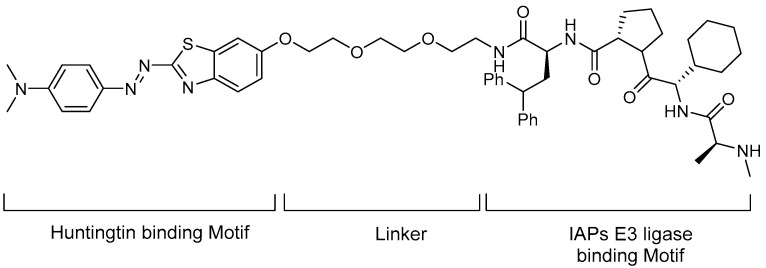
Structure of an IAP antagonist linked to a phenyldiazenyl benxothiazole derivative as an Htt degrader [35].

In 2018, the same group introduced a new Htt-PROTAC and published its mHtt degradation results [35]. Compared with the previous Htt-PROTAC, the E3 ligase was changed to IAPs and the known IAP antagonist MV1 was introduced as an E3 ligase binder. MV1 exhibits stronger affinity to cIAP1 than the bestatin analogues used in the previous Htt-PROTAC, and it also interacts with other IAPs. The new Htt-PROTAC is shown in Figure 8, in which (*E*)-2-(4-(*N*,*N*-dimethylamino)phenyldiazenyl)benzo[d]thiazole was used as POI binder and PEG linker. The authors evaluated whether the synthesized PROTAC degraded the mHtt using fibroblast cells from patients with HD and demonstrated the dose- and time-dependent reduction of mHtt.

**Figure 9:**
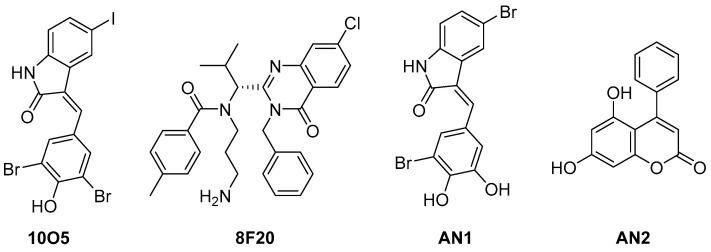
Structure of mHTT–LC3 linker hit compounds and other identified effective linker compounds as Htt degraders [37].

mHTT has been successfully degraded using ATTEC technology [36]. In 2019, Li et al. reported the discovery of mHTT–light chain 3 (LC3) linker compounds (Figure 9) that induce direct binding of mHtt and MAP 1A/1B LC3, and presented the degradation activity of mHtt through lysosome [37]. To identify small-molecule linker compounds, they performed microarray-based screening to measure the interaction of the compounds with purified human LC3B protein, a control wild-type Htt exon 1 fragment, and a pathogenic mHTT exon 1 fragment with an expanded polyQ protein with 3375 compounds. Two hits were identified from the microarray-based screening and two additional compounds were obtained by screening compounds that showed structural similarity with the hits. Allele-selective lowering of mHTT protein by HTT–LC3 linker compounds was observed in cultured primary cortical neurons, and the degradation pathway was predicted to be via autophagy. The in vivo anti-HD effects were further investigated using an HD-knock-in mouse model through two administration pathways (intracerebroventricular and intraperitoneal injections), which revealed considerable lowering of mHtt levels in the cortices of HD mice. The linker compounds 10O5 and AN2 were proven to penetrate the blood–brain barrier.

### 5.3. Alpha-Synuclein

**Figure 10:**
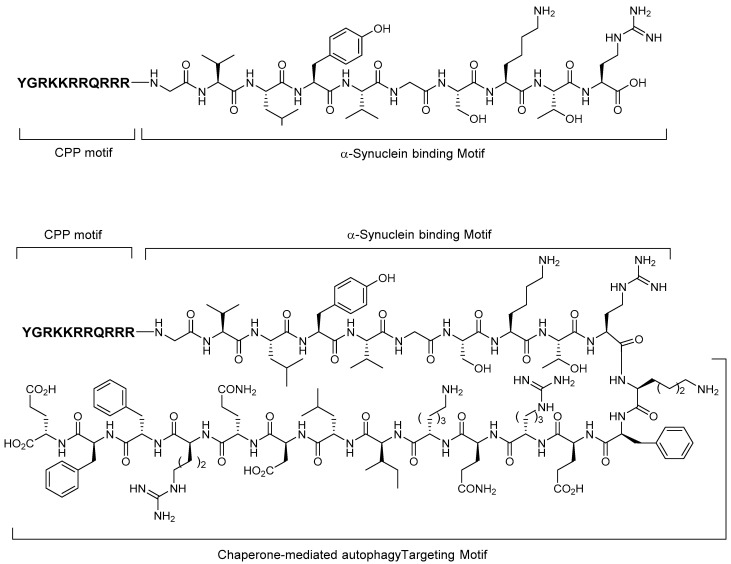
Structures of peptide-based α-synuclein degraders.

In 2014, peptide-directed lysosomal degradation of α-synuclein (18 kDa) with an appropriate peptide binder was reported by Fan et al. [38] The peptide degrader consists of three motifs: cell membrane-penetrating peptide motif (TAT-CPP, sequence: YGRKKRRQRRR) that allows peptides to cross the blood–brain barrier and plasma membrane, α-synuclein-binding motif (α-syn-BM, sequence: GVLYVGSKTR), and chaperone-mediated autophagy-targeting motif (CMA-TM, sequence: KFERQKILDQRFFE) that directs the target proteins to the lysosomal proteolytic machinery through the peptide–protein interaction (Figure 10). The authors synthesized several peptides for target protein and α-synuclein degradation, with CPP-α-synBM as a negative control compound and TAT-CPP-α-synBM-CMA-TM as a degrader. An in vitro experiment using cultured neurons demonstrated that TAT-CPP-α-synBM-CMA-TM successfully and specifically decreased the α-synuclein levels without disturbing the PSD-95 protein, whereas CPP-α-synBM slightly increased the α-synuclein level in western blot analysis.

In this study, the authors also disclosed the design, synthesis, and lysosomal knockdown efficacy of peptide degraders for the scaffolding protein PSD-95 (95 kDa) and death-associated protein kinase 1 (160 kDa). The constructs are the same as α-synuclein degraders.

**Figure 11:**
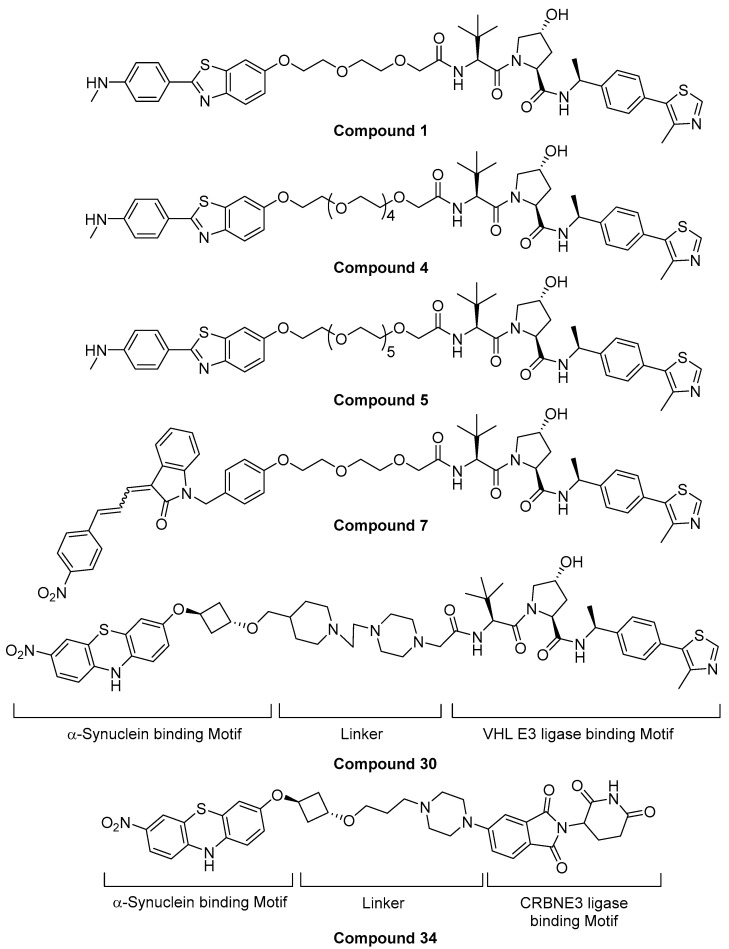
Structure of small-molecule α-synuclein degraders [39].

In 2020, a small-molecule PROTAC targeting α-synuclein protein was briefly reviewed as a “Patent Highlight” article by R.B. Kargbo [39]. The original patent was titled by “Proteolysis targeting chimeric (PROTAC) compound with E3 ubiquitin ligase binding activity and targeting alpha-synuclein protein for treating neurodegenerative diseases” by Arvinas Operations, Inc. In this article, six key α-synuclein-targeting PROTACs were selected from the patent. 2-(4-*N*-methylphenyl)benzothiazole, 1-benzyl-3-(3-(4-nitrophenyl)allylidene)indolin-2-one, and 3-nitro-10*H*-phenothiazine were introduced as α-synuclein-binding motifs, and small-molecule CRBN and VHL binders were introduced as E3 ligase binders. The two motifs were tethered by PEG or cyclic amine-based linkers. The evaluation of degradation efficacy using HEK293 T-Rex expressing A53T α-syn revealed that >65% of the target protein was degraded at 1 μM by compounds 1, 4, 5, and 34, and >30–65% of the protein was degraded at the same concentration by compounds 7 and 30 (Figure 11).

### 5.4. TDP-43-Targeting Degrader

**Figure 12:**
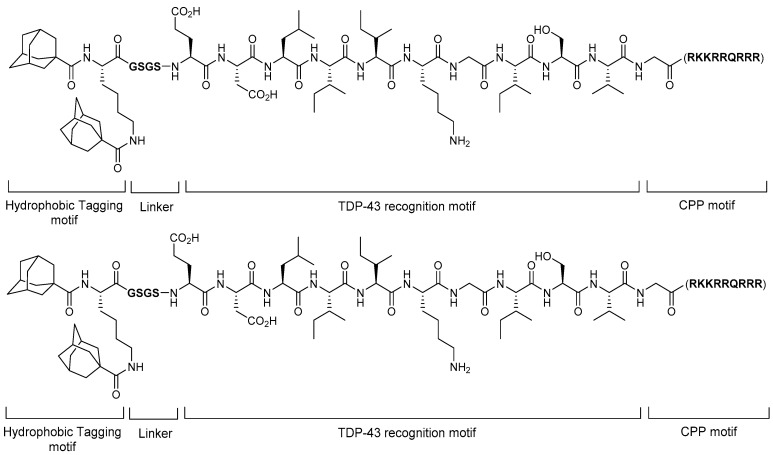
Structure of a multifunctional TDP-43 degrader with double hydrophobic tags.

Amyotrophic lateral sclerosis (ALS) is a progressive neurodegenerative disease that is currently incurable. Although the cause of ALS is not yet fully understood, TAR DNA-binding protein (TDP-43) is known to be important in ALS pathogenesis. TDP-43 belongs to the ribonucleoprotein family and is observed as ubiquitinated and hyperphosphorylated cytosolic aggregates in approximately 97% of all patients with ALS. Therefore, TDP-43 is recognized as one of the key targets in ALS. In 2019, Gao et al. reported that the HyT approach degrades the TDP-43 protein (Figure 12) [40]. They designed single and double hydrophobic-tagged TDP-43-binding peptides with cell-penetrating ability. With adamantine as a hydrophobic tag, they searched for optimal linkers between hydrophobic tags, optimal TDP-43-binding peptide sequences, and CPP sequences. Among them, the compound having di-adamantyl as a hydrophobic motif, KGSGS as a linker, EDLIKGISV as a TDP-43-binding motif, and GRKKRRQRRR as a CPP motif was found to induce TDP-43 degradation in cellular assays. Moreover, the degradation reduced the TDP-43-induced toxicity. This report is meaningful because it suggests that the HyT approach can potentially be used as an intervention for ALS.

## 6. Conclusions

Targeted protein degradation is a technology for artificially and selectively degrading intracellular target proteins using small- to intermediate-molecule compounds, which is a new drug modality that is completely different from conventional small-molecule drugs. The traditional drug development is based on the binding pockets of druggable targets. Occupation of small molecule inhibitors results in the loss of function of the target protein. However, these “occupancy-driven” pharmacology are limited by the absence of binding pockets of undruggable targets [41]. Overcoming the limitation of the “occupancy-driven” pharmacology, targeted protein degradation has shown that the loss of function of the target protein by removal of the target proteins is a result of the binding event. Because these chemical-mediated targeted protein degradation technologies work through the “event-driven” pharmacology, it has great potential to overcome problems associated with existing “occupancy-driven” pharmacology [41]. To date, several targeted protein degradation technologies have been developed, including PROTAC, molecular glues, and HyT, which achieve degradation by hijacking the UPS, as well as AUTAC, ATTEC, and LYTAC, which use the autophagy–lysosomal pathway as the degradation machinery. In particular, PROTACs have been proven to be efficient in degrading targeted proteins with >60 successful examples, two of which are currently in clinical trials focused on prostate and breast cancer treatment.

Neurodegenerative diseases have been defined as a group of intractable disorders that are characterized by progressive degeneration of neurons, resulting in neurological and psychiatric symptoms. Therapeutic targeting of protein misfolding and aggregation is currently being explored. Protein aggregates are considered to play a central role in the onset of neurodegenerative diseases [42]. Many neurodegenerative diseases are due to proteinopathies that result from aberrant protein aggregations and accumulations. To remove or prevent the formation of pathogenic protein aggregates in neurons or the brain, several approaches, such as the use of small molecules to inhibit protein production pathways, antibodies against target protein aggregates, gene silencing/suppression through RNA interference, and antisense oligonucleotides, are being intensively studied, some of which are being evaluated in clinical trials. However, the orally applicable small chemical approaches are more suitable for NDs than antibodies and oligonucleotide with potentially poor pharmacokinetic properties, although the CNS bioavailability still needs to be improved for this purpose. Cell permeability, tissue distribution, and pharmacokinetic properties can be improved using optimized cross-linkers of bifunctional molecules. Therefore, technologies for lowering the levels of pathological protein aggregates seem to have a significant potential in the treatment of neurodegenerative diseases in the future.

## Data Availability

Data sharing not applicable.
